# Ultra-thin self-healing vitrimer coatings for durable hydrophobicity

**DOI:** 10.1038/s41467-021-25508-4

**Published:** 2021-09-01

**Authors:** Jingcheng Ma, Laura E. Porath, Md Farhadul Haque, Soumyadip Sett, Kazi Fazle Rabbi, SungWoo Nam, Nenad Miljkovic, Christopher M. Evans

**Affiliations:** 1 Department of Mechanical Science and Engineering, University of Illinois, Urbana, IL USA; 2 Materials Research Laboratory, University of Illinois, Urbana, IL USA; 3 Department of Material Science and Engineering, University of Illinois, Urbana, IL USA; 4 Department of Electrical and Computer Engineering, University of Illinois, Urbana, IL USA; 5 Beckman Institute of Science and Technology, University of Illinois, Urbana, IL USA; 6 International Institute for Carbon Neutral Energy Research (WPI-I2CNER), Kyushu University, Fukuoka, Japan

**Keywords:** Polymer chemistry, Surface chemistry, Polymers

## Abstract

Durable hydrophobic materials have attracted considerable interest in the last century. Currently, the most popular strategy to achieve hydrophobic coating durability is through the combination of a perfluoro-compound with a mechanically robust matrix to form a composite for coating protection. The matrix structure is typically large (thicker than 10 μm), difficult to scale to arbitrary materials, and incompatible with applications requiring nanoscale thickness such as heat transfer, water harvesting, and desalination. Here, we demonstrate durable hydrophobicity and superhydrophobicity with nanoscale-thick, perfluorinated compound-free polydimethylsiloxane vitrimers that are self-healing due to the exchange of network strands. The polydimethylsiloxane vitrimer thin film maintains excellent hydrophobicity and optical transparency after scratching, cutting, and indenting. We show that the polydimethylsiloxane vitrimer thin film can be deposited through scalable dip-coating on a variety of substrates. In contrast to previous work achieving thick durable hydrophobic coatings by passively stacking protective structures, this work presents a pathway to achieving ultra-thin (thinner than 100 nm) durable hydrophobic films.

## Abstract (web summary)

By now a plethora of ultrathin hydrophobic coatings are available but their durability are not well developed. Here, the authors present a thin, durable and fluorine-free PDMS-based vitrimer coating that implements many desirable aspects like energy efficiency, durability and sustainability.

## Introduction

Low surface energy hydrophobic materials have the potential to enable a plethora of functions including self-cleaning^1,2^, anti-icing^3,4^, anti-fogging^5^, anti-bacterial^6^, anti-fouling^7,8^, reduced hydrodynamic drag^9^, and enhanced heat and mass transport^10^. The majority of engineering materials such as pure metals, alloys, ceramics, and semiconductors are intrinsically hydrophilic. Therefore, achieving stable hydrophobicity with these materials relies on developing hydrophobic coatings, which are commonly made from perfluoro-compounds (PFCs) due to their low surface energy (5–20 mJ·m^−2^)^11^. However, PFC coatings are not able to achieve long-term hydrophobicity (1-year long lifetime) for many applications because they lack mechanical robustness^12–15^. Recent work has shown that surface defects such as pinholes or scratches lead to significant shortening of coating lifetime. Pinhole defects lead to water penetration, which attacks the coating-substrate interface resulting in coating delamination^16,17^. Furthermore, abrasion-induced crack formation in PFC coatings results in exposure of the intrinsically hydrophilic substrate, leading to wetting and droplet pinning^12,13^. As polymeric PFCs usually have poor mechanical properties and low Young’s modulus (1–10 GPa), pinholes are easily formed on the surface leading to premature coating failure.

Without fully understanding the importance of pinhole defects, works in the past decade have attempted to enhance the overall coating robustness by added mechanical protective structures^12,13,18–20^ to act as sacrificial armor. Despite the demonstrated durability, such designs have a major drawback: the modified coatings need to be thick (coating thickness *h* > 10 μm) due to the height of the protective structures. Many applications require hydrophobic coatings to be thin (*h* < 100 nm). Some of the most important include dropwise condensation^15,21^, atmospheric water harvesting^12^, and water desalination^22^, where the heat and mass transfer rate can be enhanced by an order of magnitude by using hydrophobic surfaces, with the merit of these offset if the coating is thick (*h* > 100 nm) and thermally insulating^15,23^. Although in recent years significant effort has been placed on designing thick and thermally conductive composite materials to overcome this challenge, scalable fabrication techniques for these coatings remain elusive^14,20^. The importance of defect prevention has been increasingly realized for improving the durability of thin hydrophobic coatings. A promising path is to develop scalable, self-healing thin coatings (*h* < 100 nm) which do not necessarily need the same hardness or elastic modulus as inorganic materials but instead can enhance coating durability by actively repairing defects.

Among several available material designs for self-healing coatings, vitrimers and dynamic networks are promising for scalable thin film (*h* < 100 nm) synthesis. Commonly used self-healing materials consist of composites that embed self-healing agents in the form of microscale capsules^24^ or microvascular networks^25^ that are released upon damage. The structure of the healing agents is typically large (larger than 1 μm), must be designed to hold a range of liquids^26,27^, and would be difficult to process into thin films with thickness less than the length scale of the capsules. Vitrimers, dynamic covalent polymer networks with associative or network conserving bonds, have been demonstrated to exhibit self-healing abilities in addition to reprocessability^28–31^. The nature of the bond exchange enables vitrimers to retain their modulus upon heating^32,33^. If we consider the boric esters as the treated healing agent, their length scale approaches ~1 Å. The fabrication of vitrimer-based self-healing materials is also much more straightforward when compared to other self-healing materials. Although vitrimer chemistries have been used to achieve water repellency^34^, or self-healing^27,35–37^, no past study has succeeded in developing thin-films (*h* < 100 nm) that utilize the vitrimer chemistry for robust self-healing and hydrophobicity.

In this work, we report the design and synthesis of a vitrimer thin film with polydimethylsiloxane network strands and dynamic boronic ester crosslinks (dyn-PDMS) to take advantage of the inherent hydrophobic nature of silicones. The dynamic bonds provide a mechanism for self-healing and damage resistance. We show that even for films having nanoscale thickness (smaller than 10 nm), the transparent coating maintains exceptional hydrophobicity after scratching, cutting, indenting, and steam condensation. The long-term durability of the coating is examined by exposing the surface to steam condensation due to its sensitivity to surface defects. We demonstrate that our dyn-PDMS coating can be deposited easily on a variety of substrates using spin-coating, as well as more scalable approaches such as dip-coating. When deposited on a roughened aluminum surface, the dyn-PDMS coating also demonstrates superhydrophobicity. The PDMS vitrimer coating developed here is also relatively environmentally friendly compared to well-documented environmental and health concerns from fluorine-based chemistry^11,38,39^. In contrast to previous work that achieves thick durable hydrophobic coatings by passively stacking protective structures, our work represents a method to manufacture ultra-thin and durable hydrophobic films.

## Results

### Thin-film fabrication and characterization

**Fig. 1:**
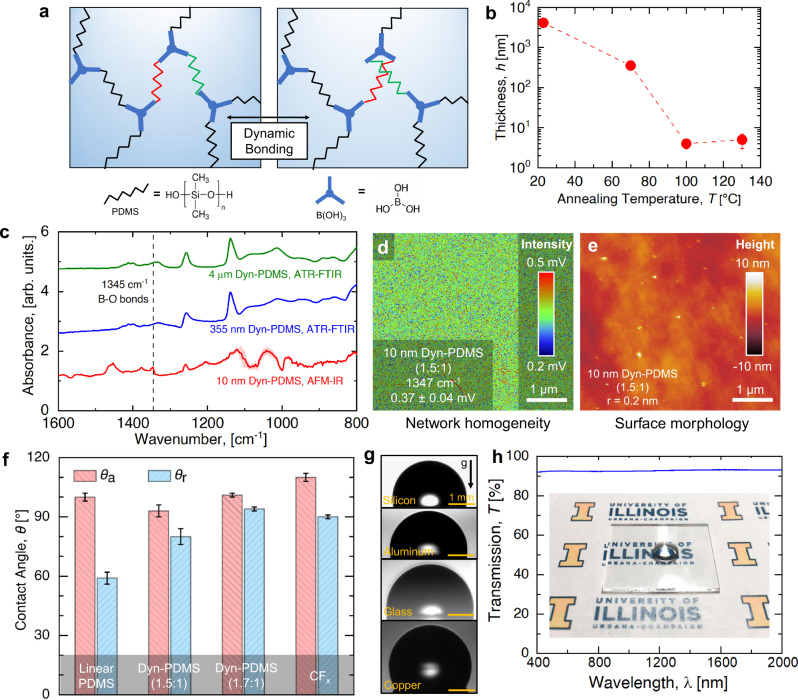
Molecular design and characterizations of dyn-PDMS. **a** Schematic and chemical structure of a dynamic network made with PDMS strands and boric acid through transesterification. Green and red strands highlight the dynamic bond exchange. **b** Correlation between the coating thickness and annealing temperature for 550 g·mol^−1^ PDMS. **c** Infrared spectra showed the B–O peak at 1330–1345 cm^−1^ for dyn-PDMS (1.5:1) films having different thicknesses. The AFM-IR spectrum was obtained by averaging 10 independent measurements and the uncertainty, shown as the red shaded area, was determined using the standard deviation of the measurements. **d** Absorbance intensity of the 1347 cm^−1^ peak on the 10-nm thin dyn-PDMS film scanned by AFM-IR. Inset color bar: IR intensity from 0.2 mV (blue) to 0.5 mV (red). **e** Surface morphology of 10-nm dyn-PDMS (1.5:1) film spun-casted on a silicon wafer. Inset color bar: surface height from −10 nm (black) to 10 nm (white). **f** Apparent advancing and receding contact angles of DI water droplets on different thin films deposited on a polished Si wafer. The thickness of the films is labeled on the bar chart. **g** Optical images of the apparent advancing contact angle on dyn-PDMS coated substrates showing substrate-independent hydrophobicity. The uncertainty of the contact angle measurements is determined from the standard deviation of 3 independent measurements at 3 different locations on one sample. **h** Transmission spectra of a dyn-PDMS coated glass slide showing high transparency. Inset: Photo image of a 2 cm × 2 cm dyn-PDMS coated glass placed on paper. The University of Illinois was printed on paper to show the transparency of the sample. A water droplet was placed on the sample to show its hydrophobicity.

The dyn-PDMS material was synthesized using poly(dimethyl siloxane) diol (Sigma Aldrich, average molecular weight = 550 g·mol^−1^, kinematic viscosity = 25 cSt) and boric acid (Sigma Aldrich, 99.5%). A stoichiometric ratio of 1.5:1 PDMS to boric acid (B(OH)_3_) was calculated and weighed out on a high precision analytical balance at gram scale. The boric acid was dissolved in isopropanol (Sigma Aldrich, 99%) in a ratio of 0.1 g·mol^−1^ B(OH)_3_ to 2.5 mL of isopropyl alcohol (IPA) by sonication at room temperature for 30 mins. The B(OH)_3_ + IPA solution was added to PDMS in a 20 mL vial which was heated at 75 °C and stirred at 250 rpm for 30 mins. The temperature was then increased to 105 °C, and the reaction vial was uncapped to proceed in an open environment. The boric acid-diol complexation results in trigonal boron sites, where the B–O bonds can exchange in an associative, or conserved, manner (Fig. 1a)^40^. Twenty minutes after bubbles formed in the solution, indicating the release of H_2_O due to the reaction between the PDMS diol and boric acid taking place and the beginning of network formation, the temperature was decreased to room temperature to prevent full conversion. We showed a critical decrease in the –OH by analysis of the FITR spectrum^40^. The initial solution has 4 mL of IPA per 1 gram of PDMS, and the IPA is then reduced due to evaporation on heating. When the IPA has been reduced from 4 mL to 1 mL, which can be gauged based on the size of the vial, then the solution is ready for spin coating. The PDMS concentration in IPA was 1 g·mL^−1^ and the viscosity was ~35 cSt. Bulk characterization of the dyn-PDMS networks showing the macroscopic modulus and stress relaxation time can be found elsewhere^40^.

To make dyn-PDMS thin films, the PDMS/B(OH)_3_/IPA solution was first strained through a 0.2 μm PTFE filter to remove impurities. Silicon wafers, glass, copper, and aluminum substrates were first cleaned by rinsing in ethanol, water, and IPA in sequence, then purified through air plasma cleaning (Harrick Plasma, PDC-32G) for ten minutes at high power (RF, 18 W). Substrates were used within 15 min of cleaning. For spun-coated samples, 0.1 mL of filtered solution was pipetted onto 2 cm^2^ substrates. The same ratio was used for larger samples. A Polos SPS Spin Coater was used with the following spin-coating procedure conditions for all samples: spin speed of 6000 rpm, spin acceleration of 500 rpm·s^−1^, test length of 150 s. For dip-coated substrates, the substrate was lowered into the PDMS/B(OH)_3_/IPA solution. The edges and backside of each substrate were dabbed dry using a Kimwipe. Note that dip-coated samples were generally thicker than samples made with spin-coating. As the deposited material by dip-coating is initially much thicker than the spun-coated material, the final material does not reduce to the same thickness as the spun coat samples under the same curing conditions.

Thin films are sensitive to the time and temperature of the drying protocol. Thin-film PDMS vitrimer samples were first cured in a vacuum oven at 100 °C for 15 h at a vacuum level of 28 inHg or 6 kPa absolute pressure. A sharp reduction in film thickness from 2 μm to about 10 nm was observed and attributed to low molecular weight PDMS diol being removed by evaporation from vacuuming prior to curing into a network. Samples made via this procedure have shown good reproducibility with a consistent thickness of 6 ± 3 nm and consistent contact angle behavior. Note that all the thickness measurements are performed using the samples on a polished silicon wafer. The sample thickness on metals is challenging to measure due to the relatively large metal surface features comparing to coating thickness. When the thin film samples were heated without vacuum on a covered hot plate at 60 °C for 3 h, followed by 100 °C heating overnight, the resulting films were ~2 μm thick. The polymer strands incorporated into the network with pre-curing are less susceptible to volatility. To better control the thickness of the developed coatings, a series of vacuum tests (all at 28 inHg vacuum level) were conducted as shown by Fig. 1b. It was determined that 12 h at 80–90 °C produced sub-10 nm thick films, while 12 h at 65 °C led to 350 ± 10 nm thick films. For the same tests done at room temperature with no heat added to the vacuum oven, the thin films were ~4 µm thick. By conducting extensive fabrication trials, an effective method for thickness control was determined by tuning the temperature of the vacuum oven during the curing step.

Vitrimer films thinner than 10 nm have not been studied, hence it was important to understand whether the PDMS-boric acid network remains intact after the thermal annealing process. We examined the existence of the B–O bonds in dyn-PDMS films of different thickness by infrared (IR) spectroscopy, as shown in Fig. 1c. For bulk materials, the distinct appearance of B–O bonds in dyn-PDMS when compared to linear PDMS has been demonstrated in previous work^40^. Attenuated total reflection Fourier-transform infrared spectroscopy (ATR-FTIR, Bruker Alpha) was used to study dyn-PDMS films having 355 nm and 4 µm thicknesses. The ATR-FTIR penetration depth was >1 µm, and showed poor sensitivity for thinner (5 nm thick) dyn-PDMS films. Therefore, photo-induced force microscopy (AFM-IR, Molecular Vista) was used to obtain better sensitivity for the 5 nm thick film. As shown by Fig. 1d, for the dyn-PDMS films, a B–O peak was observed at 1330–1359 cm^−1^ for both samples, showing the existence of B–O bonds and confirming the existence of the network. We emphasize that although a part of the IR signal of the 355 nm thick film comes from the polished silicon wafer substrate, the substrate does not contain characteristic peaks at the 1359 ± 5 cm^−1^ position, which further confirms the presence of B–O bonds. Even for the thinnest networks, the 5 nm film was shown to be spatially uniform based on a scan of the absorption peak at 1359 cm^−1^ of a 5 µm × 5 µm area (Fig. 1d). The majority of intensity variations is expected to stem from the change in surface chemistry (uncontrolled volatile organic compounds absorbed on the surface^41^) and instrument noise (usually within 30% of the intensity from multiple sources including optical forces, tip enhanced and direct thermal expansion and photoacoustic effects^42^) rather than changes in surface morphology. The AFM scan image in Fig. 1e shows the sample to be smooth, with roughness on the order of 1 nm. Furthermore, we confirmed the existence of the film and measured the thickness of the film on a polished silicon wafer using AFM step scanning, which also showed the existence of the nanoscale thin film (see Supplementary Method 1 and Supplementary Fig. 8).

To compare the mechanical robustness of the PDMS vitrimer film with PFC materials, we also deposited a 75-nm-thick amorphous fluorinated polymer film (CF_x_, x ~ 1) as a control sample and model material on the same polished Si wafer substrate using plasma-enhanced chemical vapor deposition (PECVD, See Method Section). A second control sample was made from a commercial permanent PDMS network kit (Sylgard 184, Dow Chemicals) to show the unique healing ability of the dynamic network. Further details of the material fabrication, characterizations, as well as equipment and protocols used are included in the Methods section.

### Surface chemistry and wettability

The wettability of dyn-PDMS films was determined by spin-coating on a polished Si wafer and performing water contact angle measurements using a microgoniometer (MCA-3, Kyowa Interface Science). The apparent advancing contact angle (*θ*_a_) on a flat surface largely reflects the intrinsic surface energy of the material^43^, and contact angle hysteresis, defined as the difference between advancing and receding contact angles $\triangle \theta ={\theta }_{{{{{{\rm{a}}}}}}}-{\theta }_{{{{{{\rm{r}}}}}}}$, characterizes the degree of chemical and topological homogeneity of the surface^44^. As the hydrophobicity of the PDMS vitrimers mainly stems from the contribution of the PDMS backbone, we first examined the wettability of the linear-PDMS thin film to estimate the upper-bound of the dyn-PDMS apparent advancing contact angle. For a deionized (DI) water droplet on the 17 ± 1 nm thick linear-PDMS film, we measured *θ*_a_ = 100 ± 1° which is consistent with previous results on methyl group-terminated surfaces^45^. For the same DI water droplet on the dyn-PDMS film, *θ*_a_ = 93 ± 3° (Fig. 1f) which is slightly lower than the linear-PDMS film, presumably due to the presence of hydrophilic boronic esters. Although the dynamic polymer network has a reduced *θ*_a_ compared to linear-PDMS, the contact angle hysteresis of Δ*θ* = 13 ± 4° was much smaller than for the linear-PDMS film (Δ*θ* = 40 ± 1°). We found that the high contact angle hysteresis of the linear PDMS did not originate from surface impurities or heterogeneity, but rather because the linear PDMS has small molecular size and behaves like a viscous liquid. During steam condensation on a linear PDMS coated Si wafer sample, we observed droplet cloaking (see Supplementary Fig. 3). Hence, the high contact angle hysteresis originated from droplet pinning by capillary forces from the coating material. The hydrophobicity of the surface could be further improved by tuning the ratio between PDMS and boric acid. We found that slightly increasing the PDMS to boric acid ratio from the stoichiometrically ideal 1.5:1 to 1.7:1 resulted in the highest possible advancing contact angle Δ*θ* = 100 ± 1° and a smaller contact angle hysteresis of Δ*θ* = 6° (Fig. 1f).

In order to obtain a quantitative measure of surface energy, we characterized the contact angle behavior on both 1.5:1 and 1.7:1 dyn-PDMS using a non-polar liquid, diiodomethane (Sigma Aldrich, ReagentPlus, 99%). Details of the surface energy measurements were described previously^45^. The advancing contact angles *θ*_a_ of diiodomethane droplets on 1.5:1 and 1.7:1 dyn-PDMS were 72 ± 2° and 73 ± 2°, respectively. Combined with the contact angle of deionized water droplets, the surface energy of the 1.5:1 dyn-PDMS was determined to be *γ*_s,d_ = 21.8 ± 0.6 mJ·m^−2^, and *γ*_s,p_ = 3.1 ± 0.9 mJ·m^−2^. The surface energy of the 1.7:1 dyn-PDMS was determined to be *γ*_s,d_ = 21.3 ± 0.6 mJ·m^−2^, and *γ*_s,p_ = 1.2 ± 0.7 mJ·m^−2^. The dispersive surface energy of both surfaces is consistent with measurements on a permanent PDMS surface (19–21 mJ·m^−2^)^46^. We do not expect the boric oxides to contribute significantly to the dispersive surface components because the atoms from the DMS diols are 10 times higher in concentration than the ones contributed by the boric acid, hence the van der Waals interaction between dyn-PDMS with water should be similar to typical PDMS. The total surface energy of both dyn-PDMS samples was ~23–25 mJ·m^−2^, slightly higher than commonly measured on polytetrafluoroethylene (PTFE, 10–20 mJ·m^−2^)^43^. Details of the surface energy measurements are included in Supplementary Method 2. Coatings on aluminum and copper substrates showed similar advancing contact angles with higher Δθ due to the roughnesses of these substrates (Fig. 1g, Supplementary Fig. 1, Supplementary Table 1). In addition to dyn-PDMS’s hydrophobic property, it is transparent as shown by UV–Vis-NIR spectra (Varian Cary 5G UV–Vis-NIR Spectrophotometer) in Fig. 1h, and can be applied on solar panels or commercial windows to achieve self-cleaning, in contrast to many other PFC-based thick coatings^13^. Details of the contact angle experimental setup and methodology are included in the Methods section. The apparent contact angles, steam condensation performance, and surface morphologies of multiple dyn-PDMS films are included in Supplementary Figs. 1, 2, and 5, respectively.

### Self-healing properties

**Fig. 2:**
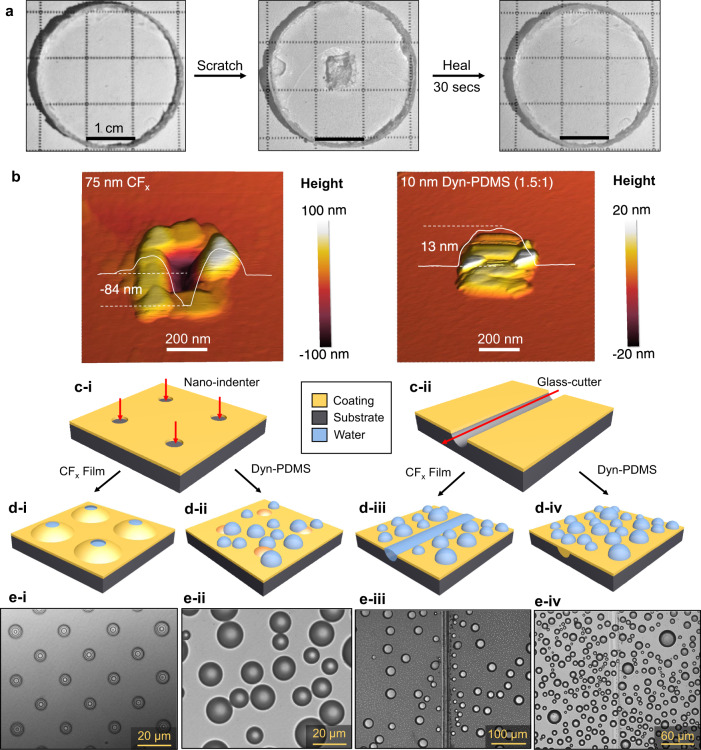
Self-healing of dyn-PDMS films. **a** Optical top-view images of a bulk dyn-PDMS cylinder healing at room temperature after being scratched in the center using a razor and healed at room temperature after 1 psi of pressure was applied. **b** Response of the materials to AFM area scratches with a contact force of 2 μN. The area scratch was performed at the center 200 nm × 200 nm area, and left permanent damage on CF_x_ (left image), while the self-healing dyn-PDMS film (right image) was left with a bump of materials covering the damaged hole. Inset color bar: surface height from low (black) to high (white). **c** Comparison of dyn-PDMS and CF_x_ thin-film hydrophobicity. Schematic showing the observed response of the coating to (**c**-i) artificial nano-indented pinholes and (**c**-ii) scratches. During steam condensation, optical microscopy images revealed that (**d**-i and **e**-i) water blisters formed beneath pinholes on the CF_x_ film, while (**d**-ii and **e**-ii) no blisters formed on the dyn-PDMS film. Top-view optical microscopy images showed (**e**-iii) droplets pinned near the linear water film on the CF_x_ coating after scratching, while (**e**-iv) the scratch on the dyn-PDMS sample recovered and healed as evidenced by droplets nucleating directly on the scratch without signs of pinning.

To gain experimental insight into the PDMS vitrimer’s self-healing capacity when exposed to external mechanical damage, area scratches were imposed at length scales ranging from millimeters down to nanometers to represent a variety of possible damage mechanisms. Two millimeter-thick bulk dyn-PDMS samples^40^ were synthesized and scratched using a razor blade (Fig. 2a). After pressing the damaged samples in a hydraulic press at ~1 psi gauge pressure for 30 s at room temperature, the scratches fully healed, and the sample became optically clear and as uniform as prior to damage (Fig. 2a). For thin dyn-PDMS films after thermal annealing, nanoscale defects such as pinholes and particle scratches are as critical for durability as macroscale damage^16^. Hence, we also examined the self-healing performance at the nanoscale by creating scratches and pinholes using AFM. A constant force of 2 μN was applied by the AFM tip to the surface in contact mode when making an area scratch (200 nm × 200 nm area). Tapping mode imaging was then performed in situ one minute after damaging the surface. We observed that material surrounding the area scratch re-combined, and an ~10 nm tall bump formed, completely covering the pinhole (Fig. 2b). We were unable to observe the dynamic process of healing using AFM as the pinhole was already covered by material in the time it took to scan the 200 nm by 200 nm area (10–100 s). The precise molecular mechanism governing the nanometer-scale self-healing is under current investigation; however, the sample viscosity is close to 10^7^ Pa·s at room temperature which is too large for gravity-driven flow on these time scales^33^. The rapid healing response facilitated by the dynamic bonds and promoted by the thin layer of material was qualitatively different when compared to the PFC polymer thin films. Applying the same area damage to the surface of the 75 nm-thick CF_x_ film deposited on an identical Si wafer substrate left permanent scratches and pinholes that could not be removed (Fig. 2b).

The self-healing capacity of our dyn-PDMS thin film enables the prevention of degradation in hydrophobicity caused by defects and mechanical damage from pinholes and scratches. Specifically, nanoscale pinholes that are commonly observed on thin polymer films lead to moisture penetrating and reaching the coating-substrate interface, which results in water blister formation and delamination^16,45^. The continual delamination eventually exposes the hydrophilic substrate, representing the key limitation for the successful development of thin and durable hydrophobic coatings for condensation applications over the past century^15,17,47^. As demonstrated in Fig. 2(c-i), we created artificial pinhole arrays on CF_x_ thin films using nanoindentation at 1.2 mN force with a standard Berkovich tip and observed the behavior of steam condensation on these pinholes using a top-view optical microscope (See Method Section). Steam condensation on the pinholes results in arrays of water blisters forming beneath the coating. However, using the same indentation method on our dyn-PDMS film did not result in visible pinhole formation due to rapid self-healing. Hence, no blisters were able to initiate while steam was condensing on the sample (Fig. 2d-ii, 2e-ii).

Scratch defects are another critical and well-known limitation to achieving durable hydrophobicity. Water droplets having contact with scratches are pinned in place which results in water film formation due to adhesion with the exposed hydrophilic substrate. We conducted scratch experiments on both dyn-PDMS and CF_x_ films using a glass cutter to damage both the coatings and the polished Si wafer beneath the film. When condensing steam on the CF_x_ sample, the scratch-exposed hydrophilic Si wafer initiated local water formation in the scratch in the form of a thin linear film that filled the scratch and advanced on the hydrophobic areas due to Gibbs criteria-initiated pinning^48^ (Fig. 2d-iii, 2e-iii, also see Supplementary Video 1). However, on the dyn-PDMS surface discrete droplets grew over the damaged scratch and were not pinned, showing that the damaged area was able to recover its hydrophobicity (Fig. 2d-iv, 2e-iv, also see Supplementary Video 2).

### Applications of dyn-PDMS

**Fig. 3:**
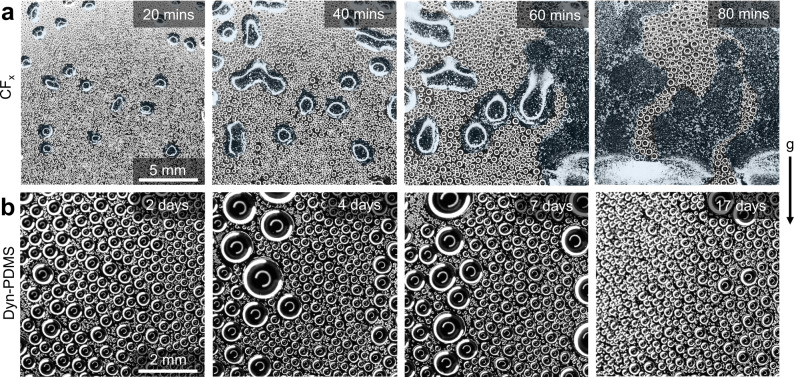
Coating durability during steam condensation. Time-lapse optical images of steam condensation on a (**a**) CF_x_ coated Si wafer and (**b**) 10 nm dyn-PDMS (1.5:1) coated Si wafer. Water blisters grew beneath the CF_x_ film and resulted in coating delamination. The water blisters in **a** are false-colored with light blue to aid in visualization. The stable dropwise condensation lasted more than 17 days on the dyn-PDMS film, which did not fail during the entire experimental run.

The demonstrated exceptional self-healing capability of our dyn-PDMS thin films makes them a suitable ultra-thin coating for long-term hydrophobicity. Due to the self-healing mechanisms of vitrimers, our dyn-PDMS thin films can resist mechanical degradation or repair early-stage pinholes and thus maintain long-term hydrophobicity. As a quantification of durability, we demonstrated long-term performance in direct steam condensation conditions and compared with conventional PFC materials. We conducted tests in a well-controlled environment capable of steady steam condensation (see Methods section). Samples were attached to a Peltier-based thermal plate vertically using double-sided copper tape. The cold plate was held at 5 °C and all experiments were done in ambient conditions of 22 °C, 45% relative humidity. The thickness difference between CF_x_ and dyn-PDMS film does not cause a difference in sample subcooling as both are ‘thermally thin’ compared to the 525 μm thick silicon wafer substrate. Hence we do not expect the subcooling difference between the two samples to be higher than 5%. We assume the thermal conductivity of the polished silicon wafer to be *k*_Si_ = 140 W·m^−1^·K^−1^, and the thermal conductivity of both the CF_x_ film and dyn-PDMS film to be ${k}_{{{{{{\rm{C}}}}}}{{{{{{\rm{F}}}}}}}_{{{{{{\rm{x}}}}}}}}={k}_{{{{{{\rm{dyn}}}}}}}$ = 0.2 W·m^−1^·K^−1^, which is the typical thermal conductivity of amorphous polymeric materials^49^. The relative thermal resistance of the CF_x_ film (${R}_{{{{{{\rm{s}}}}}},{{{{{\rm{C}}}}}}{{{{{{\rm{F}}}}}}}_{{{{{{\rm{x}}}}}}}}$) when compared to the one of polished silicon wafer (${R}_{{{{{{\rm{s}}}}}},{{{{{\rm{Si}}}}}}}$) is: ${R}_{{{{{{\rm{s}}}}}},{{{{{\rm{C}}}}}}{{{{{{\rm{F}}}}}}}_{{{{{{\rm{x}}}}}}}}/{R}_{{{{{{\rm{s}}}}}},{{{{{\rm{Si}}}}}}}=({h}_{{{{{{\rm{C}}}}}}{{{{{{\rm{F}}}}}}}_{{{{{{\rm{x}}}}}}}}/{h}_{{{{{{\rm{Si}}}}}}})({k}_{{{{{{\rm{Si}}}}}}}/{k}_{{{{{{\rm{C}}}}}}{{{{{{\rm{F}}}}}}}_{{{{{{\rm{x}}}}}}}})\approx 4$%, while the dyn-PDMS film is: ${R}_{{{{{{\rm{s}}}}}},{{{{{\rm{dyn}}}}}}}/{R}_{{{{{{\rm{s}}}}}},{{{{{\rm{Si}}}}}}}=({h}_{{{{{{\rm{dyn}}}}}}}/{h}_{{{{{{\rm{Si}}}}}}})({k}_{{{{{{\rm{Si}}}}}}}/{k}_{{{{{{\rm{dyn}}}}}}})\approx$ 0.5%, confirming the negligible difference in sample sub-cooling. As shown in Fig. 3, stable dropwise condensation occurred on the dyn-PDMS films for 17 days with no signs of degradation as quantified by the lack of formation of water blisters or increased contact angle hysteresis after conducting the full test. The apparent advancing contact angle of the tested dyn-PDMS sample was 94 ± 3° with a contact angle hysteresis of 10 ± 5°. The CF_x_ film degraded in hours due to the formation of water blisters. Over 50% of the PFC-based film delaminated after only 80 mins of condensation. Note that the water blisters shown in Fig. 3a look similar to condensate films forming on hydrophilic surfaces. To prove that the false-colored irregular-shaped objects are indeed water blisters and not films, we conducted additional optical imaging analysis which has been included in Supplementary Discussion 1. The dyn-PDMS thin films far surpassed the mechanical durability of the conventional thin films used. In addition, we also demonstrated that dyn-PDMS has good chemical robustness when immersed in organic solvents, salt water, and distilled water (see Supplementary Method 3 and Supplementary Figs. 7 and 9).

**Fig. 4:**
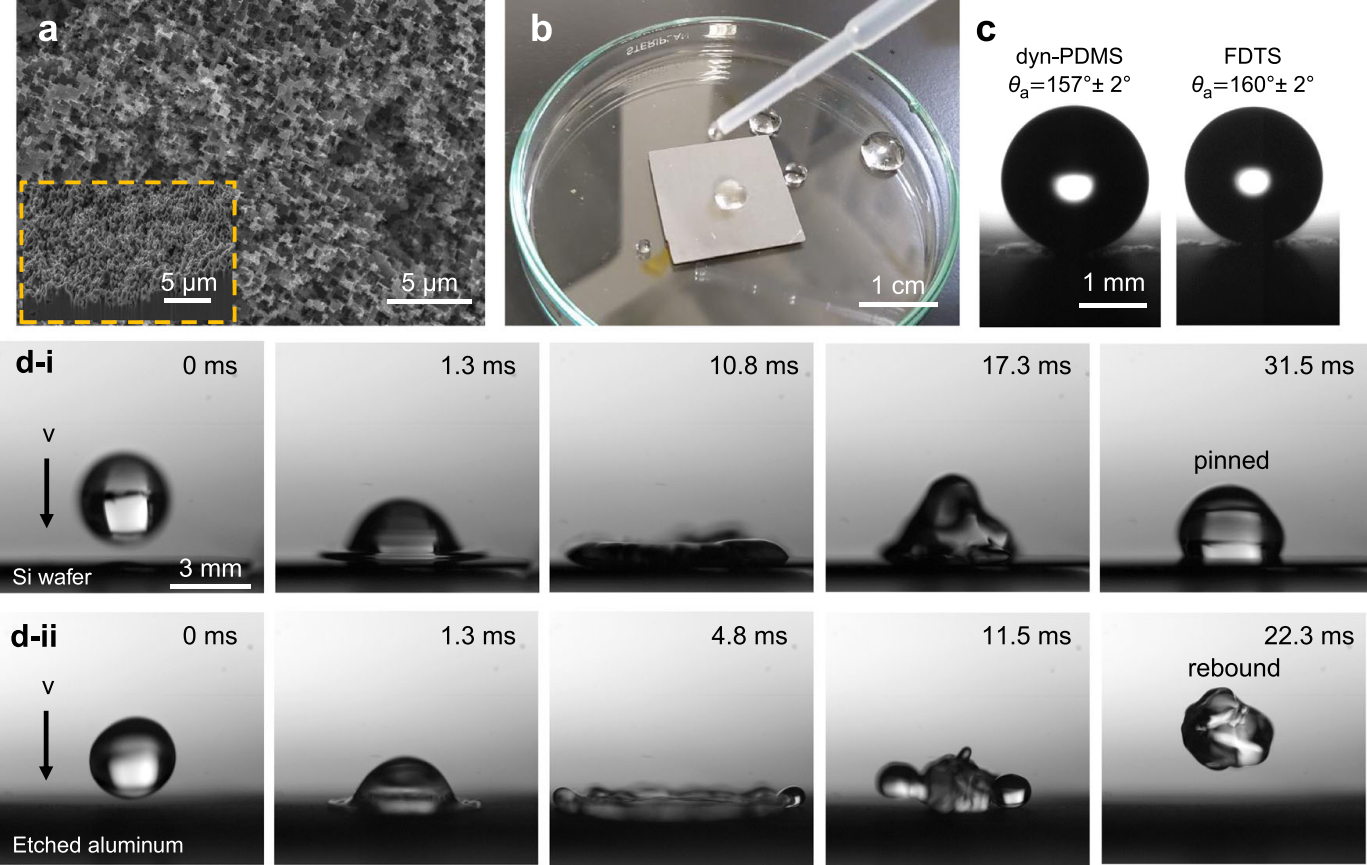
Superhydrophobicity of dyn-PDMS deposited on etched aluminum substrate. **a** SEM image of etched aluminum substrate. (inlet: cross-section SEM image). **b** Photographs of dropping water droplets on 2 cm × 2 cm sized dyn-PDMS coated etched aluminum plate. **c** Comparison between the wettability of dyn-PDMS and FDTS modified etched aluminum. **d** Time-lapse images of water droplet impacting on (**d**-i) dyn-PDMS coated silicon wafer and (**d**-ii) dyn-PDMS coated etched aluminum. Gravity points downwards in all images showing in **c** and **d**.

Dyn-PDMS can also be utilized to fabricate superhydrophobic surfaces (*θ*_*a*_ > 150°) on textured substrates by a variety of techniques like spin-coating and dip-coating. To demonstrate this, we designed a superhydrophobic surface by spin-coating the dyn-PDMS solution with a receding contact angle above 90° (1.7:1 dyn-PDMS) onto a hydrochloric acid etched aluminum substrate, which has micron-scale roughness (Fig. 4a, b). The etched aluminum substrate was chosen for its wide applicability and straightforward and scalable fabrication. The advancing contact angle of the sample was measured by microgoniometer, which gives *θ*_a_ = 157 ± 2° with hysteresis <5°. We compared the wettability of dyn-PDMS and fluorinated-silane modified etched aluminum (Methods section) and found their advancing contact angle was almost identical, as shown in Fig. 4c. This indicates that, although PFC materials have higher hydrophobicity on flat surfaces, using a textured substrate brings PFC and dyn-PDMS materials to comparable contact angles. Water droplets show high mobility on the superhydrophobic dyn-PDMS surface compared to the flat hydrophobic dyn-PDMS surface through a droplet impacting experiment (Methods section). Upon a DI water droplet (radii = 1.7 mm) impact on both surfaces at a speed of 1.3 m/s, the droplet was pinned on the flat hydrophobic dyn-PDMS surface and unable to bounce back. (Fig. 4d–i). The droplet impacted on the superhydrophobic dyn-PDMS surface completely bounced back and left the surface free of any water. (Fig. 4d–ii).

## Discussion

In addition to its demonstrated robustness, the dyn-PDMS film is also fluorine-free, which leads to greater environmental friendliness owing to lower bioaccumulation when compared to PFCs, which can take hundreds of years to degrade^11^. Silicone-based materials can be recycled and reused using organic solvents such as toluene^40^. From an application perspective, our developed PDMS vitrimer coating process is an important aspect to consider. The film can be deposited by spin-coating and also with more scalable dip-coating. One advantage of the deposition process is that the annealing step removes excessive coating materials, hence even techniques such as spray and brush coating can achieve thin layers of dyn-PDMS. The surface morphologies of spun-coated and dip-coated films on a variety of substrates scanned by AFM, as well as condensation performance, are detailed in Supplementary Fig. 5 and Supplementary Table 1.

The ultra-thin vitrimer coatings developed here not only offer a solution towards sustainable hydrophobicity, but also raise open scientific questions within materials science and fluid mechanics that remain to be answered. From a materials perspective, vitrimeric ultra-thin films have yet to be reported. The role of nano-confinement on the viscoelasticity of the materials, as well as the bond exchange rate in nanometer thin films compared to bulk materials, needs further investigation. There is a large body of work on polymer films which show deviations in physical and dynamic properties when their thickness is below a critical length scale which can vary from 2 to 200 nm^50,51^. At present, it is unknown if the dynamics or modulus are altered in the dyn-PDMS films. From a fluids perspective, the fundamental vitrimer-water interaction needs to be elucidated as dynamic networks differ from commonly used elastic solids or viscous lubricants. For example, cloaking of droplets is a common concern when using viscous liquids to achieve hydrophobicity with lubricant-infused surfaces^52,53^. The lubricant can flow over the droplet and then drain and degrade as droplets shed from the surface^54^. The cloaking criterion has been mainly considered from a thermodynamic perspective that is a function of the interfacial tensions of the system^52^. Our experiments did not observe cloaking when the coating was thin, indicating that cloaking may be limited by the total amount of coating material on the surface. The work reported here provides important design guidelines for thin vitrimer film development, not only for hydrophobic coatings, but also a variety of other durable and functional interfaces.

## Methods

### Deposition of CF_x_ thin films

We deposited conformal fluorine polymer CF_x_ (x ~ 1) on a Si substrate by PECVD using Ar/C_4_F_8_ as a feeding gas. Prior to PECVD, we prepared the Si substrates by rinsing sequentially with acetone, IPA, water, and IPA, followed by drying with a clean nitrogen gas stream. Finally, the samples were cleaned by O_2_/Ar plasma inside a chamber (Plasmatherm SLR 770, ICP) to remove any adsorbed hydrocarbons on the surface. The CF_x_ deposition was then performed inside the same chamber at a pressure of ≈20 mTorr and at ≈50 °C for 15 s. More information on the FT-IR and XPS spectrum can be found at our previous work^16^.

### Thin Film Thickness measurement

The thickness of the thin films was measured by spectroscopic ellipsometry on a J.A. Woollam VASE instrument. Spectra were collected from 300 to 1000 nm wavelengths. Film thickness was determined using the following optical model: layer 1 = Si (substrate), layer 2 = SiO_2_ (thickness = 2 nm), layer 3 = Cauchy model (A, B, C and thickness = free parameters). The thickness of layer 3 corresponds to the thickness of the polymer film. We used a Cauchy model to determine the refractive index *n* of the CF_x_ layer with respect to wavelength *λ* (in μm): $n\left(\lambda \right)=A+B/{\lambda }^{2}+C/{\lambda }^{4}$. Optical parameters, *A* = 1.37, *B* = 0.03, *C* = 0 were initially taken. For dyn-PDMS film, optical parameters, *A* = 1.5, *B* = 0, *C* = 0 were initially taken. The thickness of the 10-nm dyn-PDMS film was also determined by AFM step scanning. We made a step in the film by first adhering Kapton tape to the polished silicon wafer, then treating the surface by chemical cleaning and plasma cleaning. We then spun-coated the network solvent on the sample. The tape was peeled off after the thermal annealing of the film, and an AFM scanning was performed at the peel section, which yields a coating thickness of *h* ≈ 4 ± 3 nm. The relatively high uncertainty originates from the experimental difficulty to make a clean step, considering that the coating is able to flow to some extent. However, the thickness and IR spectrum mapping ensured the presence of the film. More information regarding the AFM scanning is included in Supplementary Method 1.

### Fabrication of etched aluminum substrate

The as-purchased aluminum surfaces (Aluminum 6061, McMaster-Carr) were first cleaned with acetone, ethanol, IPA and de-ionized water. For creating microscale roughness, the surface was immersed in 2 M Hydrochloric acid for 15 min and rinsed with de-ionized water afterward. The 2 M Hydrochloric acid was prepared by diluting as purchased 37% w/w Hydrochloric acid (HCl, Sigma-Aldrich, CAS# 7647-01-0) with de-ionized water.

### Fabrication of superhydrophobic dyn-PDMS sample

A solution of 1.7:1 PDMS to boric acid was prepared by dissolving the boric acid in 3 mL of IPA and mixing with the 1 mL PDMS in a 20 mL vial at 60 °C. The solution was then stirred at 110 °C on a hot plate and most IPA evaporated. When the solution in the vial decreased to 2 mL, the solution was in a 1:1 ratio of PDMS to IPA. The etched aluminum substrate was washed sequentially with ethanol/water/IPA and blown dry. 150 µL of 1.7:1 dyn-PDMS solution was pipetted onto the 2 cm^2^ substrate and spun coat following the same procedure as all above samples. The substrate was the cured in a vacuum oven at 110 °C for 17 h.

### Fabrication of fluorinated-silane modified etched aluminum sample

1H,1H,2H,2H-Perfluorodecyltrimethoxysilane (FDTS, CAS# 83048-65-1, Dow Corning Co.) was deposited on etched aluminum surfaces using vapor-phase deposition. Samples were first rinsed with acetone, IPA, and de-ionized water, dried in a clean nitrogen (N_2_) flow, and cleaned with plasma cleaner. Then, samples and a 50 mL beaker containing 1 mL of FDTS toluene solution (5% v/v) were sealed in a glass container and heated in an atmospheric pressure oven (Lindberg Blue M) at 80 °C for 3 h. This process allowed for the development of a highly conformal (monolayer thick) silane layer on surfaces.

### Atomic force microscopy (AFM) scanning and scratching

The developed surfaces were scanned by AFM (Cypher, Asylum Research) in air tapping mode using a cantilever with a tip radius <10 nm (Tap300AL-G, TED PELLA, INC). For sample scratching, we used the same tip in contact mode, and the force applied to the tip (*F*) was controlled by the applied voltage (*U*) with a calibrated correlation of *F*(nN) = 600*U*(V). To create line scratches, the tip was moved at a speed of 1 μm/second. Area scratches were made by scanning the area (200 nm × 200 nm) of interest with a resolution of 256 × 256 at contact mode at a frequency of 1 Hz.

### ATR-FTIR characterization

Substrates comprising of Si wafers having 3 mm × 3 mm sizes were coated with various thin films and placed on top of a diamond crystal in the Single Reflection ATR setup of the ALPHA Bruker FT-IR Spectrometer with Platinum ATR module. All samples were around 600 µm thick and held in place by the clamp mechanism. The pure diamond crystal is brazed into tungsten carbide hard metal which allows for high clamping pressures. The spectral range for the FTIR Spectrometer used was 8000 cm^−1^ to 10 cm^−1^.

### AFM-IR characterization

In AFM-IR (Molecular Vista), light-matter interaction of the structure can be captured by an AFM cantilever coupled with a light source. A tunable infrared laser (quantum cascade laser, QCL) is incident at the tip-sample interaction, thus inducing a plasmonic effect at the metal-coated tip apex (ppp-NCHAu from Nanosensors TM, spring constant = 42 N·m^−1^) and creating a lightning rod effect. Therefore, a highly concentrated electromagnetic wave can be induced on the target structure. The photo-induced force is the dipole-dipole interaction between a polarized particle (or atom) on the surface with the image on the tip apex. The near-field optical absorption is captured by using suitable lock-in amplifiers. The cantilever is modulated at the second harmonic at ~1.5 MHz and the laser is modulated at a frequency, *f*, where *f* is the difference between the first and second harmonic of the cantilever. The optical response is collected at the first harmonic (~250 kHz) of the cantilever.

### Nanoindentation

Pinholes in 6 × 6 arrays on dyn-PDMS and CF_x_ thin films were fabricated by nanoindentation (Hysitron TI 950 TriboIndenter). The distance between each pinhole was roughly 30 µm and the depth of each indent was controlled by applying a force of 1.2 mN from the indenting tip (Berkovich TI-0039 standard tip, Bruker) to the samples. The average indent depth on the CF_x_ surface was 70 ± 10 nm and on the dyn-PDMS surface was 25 ± 5 nm. Both indents were deeper than coating thickness to ensure the coating was fully penetrated.

### Long-term condensation testing

The samples were attached to a Peltier stage (TP104SC, Instec) oriented vertically using double side copper tape. The stage was connected to a temperature controller (mK2000, Instec) that was set to 5.0 ± 0.1 °C. All condensation experiments were done in ambient conditions (22 °C, 45% relative humidity). A Canon EOS Rebel T6 camera with macro lens (Canon MP-E 65 mm F/2.8 1–5×) captured images of the condensing water droplets at a 2592 × 1728 pixel resolution. The sample was illuminated with an LED ring light (LED-144-YK, AmScope).

### Condensation observation with the optical microscope

A top-view optical microscope (Nikon Eclipse LV100) coupled to a monochrome camera (Nikon DS-Qi2) was used to study water droplet condensation and evaporation in a laboratory environment having air temperature *T*_a_ = 22 ± 1 °C and a relative humidity RH = 45 ± 5% (RO120, Roscid Technologies). We placed the samples horizontally on a cold stage (Linkam T95-PE) and reduced the stage temperature to *T*_c_ = 5.0 ± 0.1 °C to initiate and observe droplet growth.

### Droplet impact experiment

The deionized water for the impacting experiment was supplied to a 25 gauge needle from a gravity bag (Enteral Feeding Gravity Bag, Dynarex) attached to the ceiling of the room. Droplets having radii ≈1.7 mm formed at the tip of the needle and impacted the substrate from a height  ≈ 10 cm, resulting in an impact speed of *v* = 1.3 m/s (absolute velocity). A high-speed camera (Phantom v711, Vision Research), coupled to a 1–5× macro lens (Canon MP-E 65 mm F/2.8 1–5×), recorded the impacting droplets at frame rates of 4000 frames per second (fps) and resolutions of 1024 × 768. A fiber-optic cable connected to a light source (EKE 150 W, Kramer Scientific) provided sufficient back-lighting to achieve an exposure time of 10–30 μs.

## Supplementary information

Supplementary Info PDF

Supplementary Info

Description of Additional Supplementary Files

Movie S1

Movie S2

## Data Availability

The authors declare that full experimental details and characterization of materials are available in the Supplementary Information. All raw data that support the findings in this study are available from the corresponding authors upon request.
