# Supplementary material for: Ultra-thin self-healing vitrimer coatings for durable hydrophobicity
Source: Nat Commun. 2021 Sep 1;12:5210. doi: 10.1038/s41467-021-25508-4 (PMC8410847; doi:10.1038/s41467-021-25508-4)
Supplement: Supplementary file 2 — Supplementary Info [file 41467_2021_25508_MOESM2_ESM.docx]

Supplementary Information for

**Ultra-thin self-healing vitrimer coatings for durable hydrophobicity**

*Jingcheng Ma^1+^, Laura E. Porath^2,3+^,* *Md Farhadul* *Haque^1^, Soumyadip Sett^1^, Kazi Fazle Rabbi^1^, SungWoo Nam^1,2,3^, Nenad Miljkovic^1,2,4,6*^, Christopher M. Evans^2,3,5*^*

^1^Department of Mechanical Science and Engineering, University of Illinois, Urbana, IL, USA

^2^Materials Research Laboratory, University of Illinois, Urbana, IL, USA

^3^Department of Material Science and Engineering, University of Illinois, Urbana, IL, USA

^4^Department of Electrical and Computer Engineering, University of Illinois, Urbana, IL, USA

^5^Beckman Institute of Science and Technology, University of Illinois, Urbana, IL, USA

^6^International Institute for Carbon Neutral Energy Research (WPI-I2CNER), Kyushu University, 744 Motooka, Nishi-ku, Fukuoka 819-0395, Japan

^+^Equal Contribution

*Corresponding Authors:

Nenad Miljkovic: [nmiljkov@illinois.edu](mailto:nmiljkov@illinois.edu) or Christopher Evans: [cme365@illinois.edu](mailto:cme365@illinois.edu)

**~~S1.~~** ~~Contact angle of dyn-PDMS (1.5:1) on different substrates~~

**~~S2.~~** ~~Optical microscopy image of steam condensing on the sample surfaces~~

**~~S3.~~** ~~Atomic force microscopy images of the sample surfaces~~

**~~S4.~~** ~~Surface energy measurements~~

**~~S5.~~** ~~Chemical robustness of the dyn-PDMS thin films~~

**~~S6.~~** ~~Sample thickness measurement using IR-AFM~~

**~~S7.~~** ~~Swelling of bulk dyn-PDMS (1.5:1) in water, hexanes and toluene~~

**~~S8.~~** ~~CF~~_~~x~~_ ~~film degradation~~

**~~Supplemental Video S1.~~** ~~Steam condensation on a scratched 75 nm thick CF~~_~~x~~_ ~~film (738 μm × 490 μm view, playing at 4× normal speed)~~

**~~Supplemental Video S2.~~** ~~Steam condensation on scratched 10 nm thick dyn-PDMS (738 μm × 490 μm view, playing at 4× normal speed)~~

**Supplementary Figures**


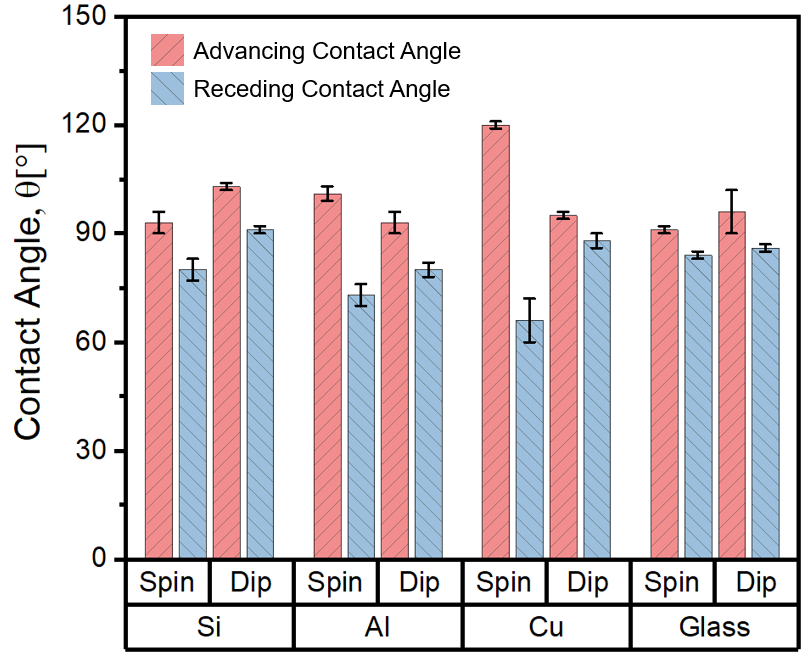


**Supplementary Figure 1.** Apparent advancing and receding contact angles of deionized water droplets deposited on dyn-PDMS films. The films were deposited on a polished silicon wafer, aluminum tab, copper tab, and glass slide using spin-coating and dip-coating. The uncertainty used for each value represents the standard deviation of three spatially distinct measurements on each individual sample.


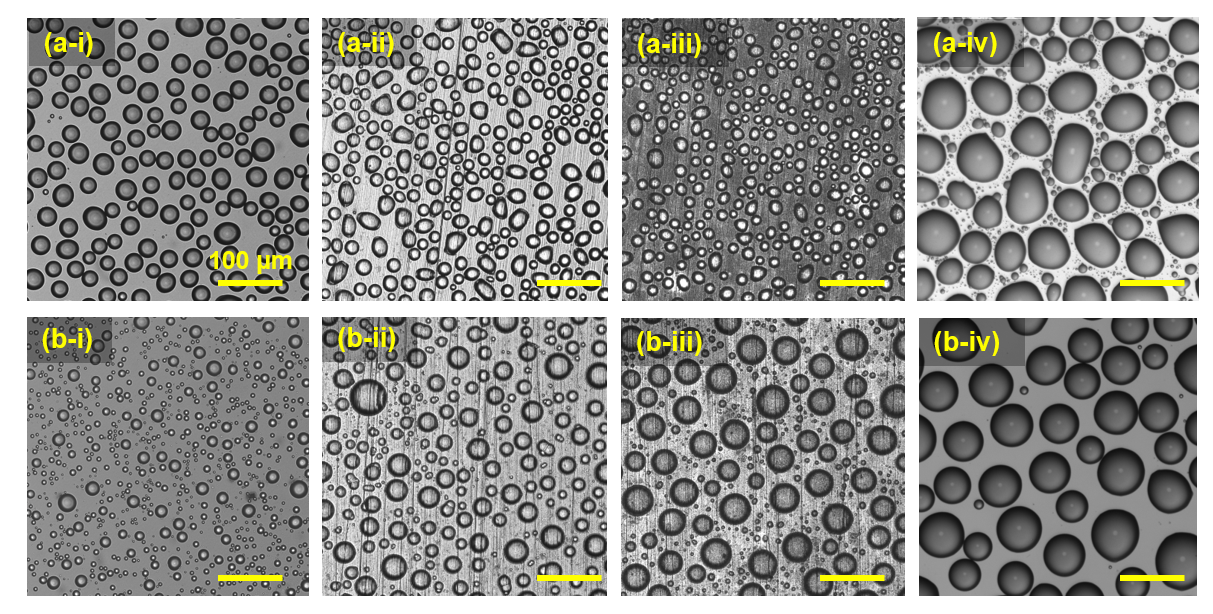


**Supplementary Figure 2.** Bright field optical microscopy of water vapor condensation on 1.5:1 dyn-PDMS coatings. The films were fabricated using (a) spin-coating and (b) dip-coating. The substrates were a (i) polished Si wafer, (ii) aluminum tab, (iii) copper tab, and (iv) glass slide. Gravity points into the page for each image.


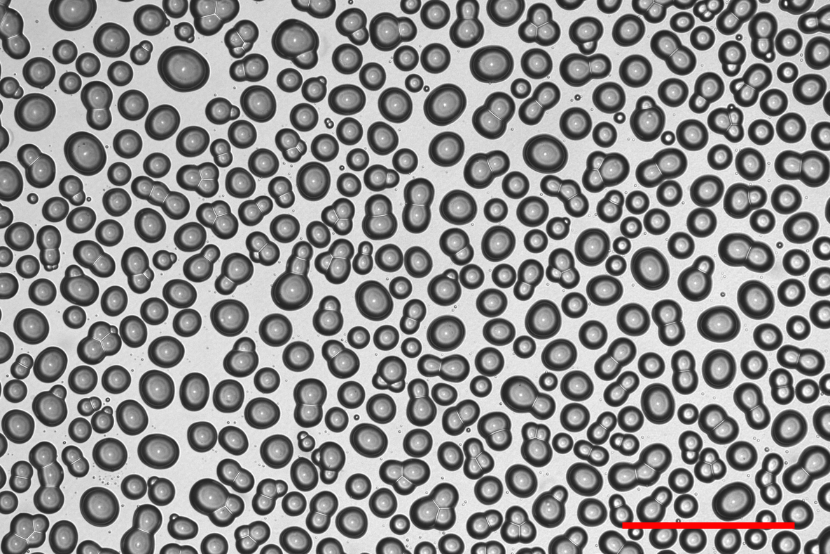


**Supplementary Figure 3.** Bright field optical microscopy image of water vapor condensation on a 17 nm-thick linear PDMS film coated Si wafer. The red scale bar in the image represents 150 μm. Gravity points into the page.


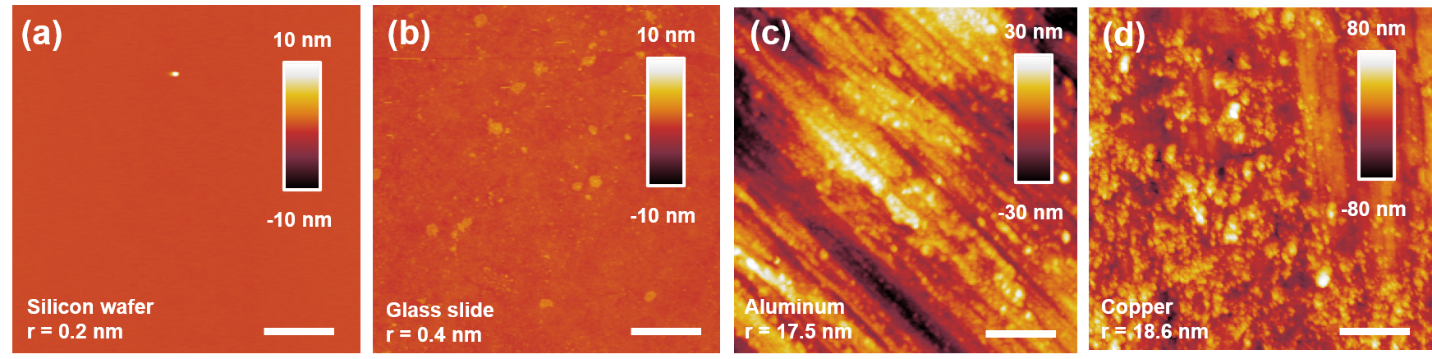


**Supplementary Figure 4.** Atomic force microscopy (AFM) images of substrates, including the polished (a) silicon wafer, (b) glass slide, (c) aluminum tab and (d) copper tab. The sample information is labeled at the lower-left corner of each image. The scale bars in all figures represent 1 μm. Inset color bar: surface height from low (black) to high (white).


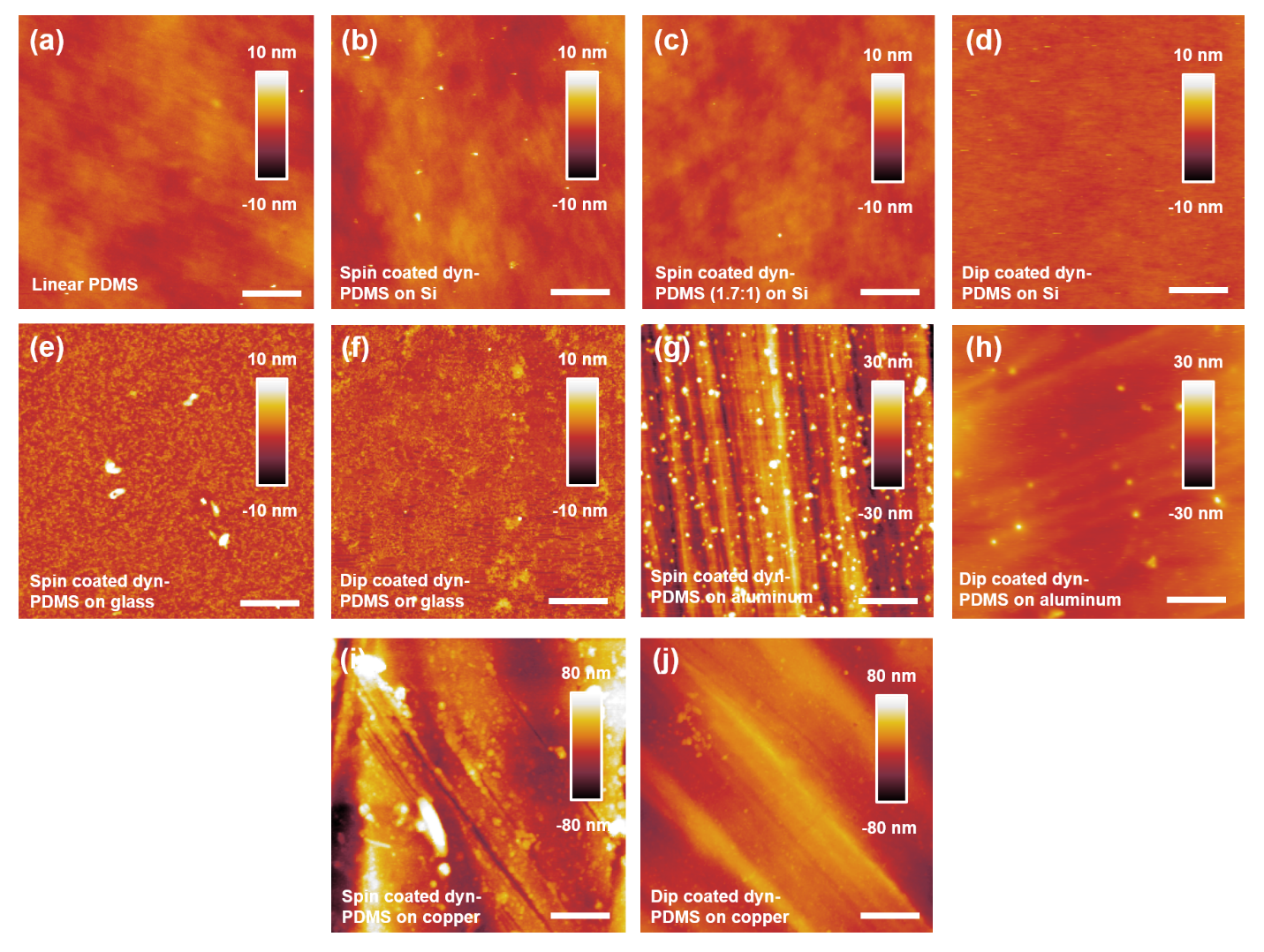


**Supplementary Figure 5.**  Atomic force microscopy (AFM) images of different films deposited on different substrates using spin-coating or dip-coating. The sample information is labeled at the lower-left corner of each image. The scale bars in all figures represents 1 μm. Inset color bar: surface height from low (black) to high (white). Dip-coating samples are generally thicker than spin-coated samples. This is because that for dip coating, the amount of materials on the substrate prior to thermal annealing is more than the ones of spin-coated samples, hence the thermal annealing recipe that can remove all excessive material for the spin-coated samples cannot guarantee the removal of all excessive materials for the dip-coated samples.


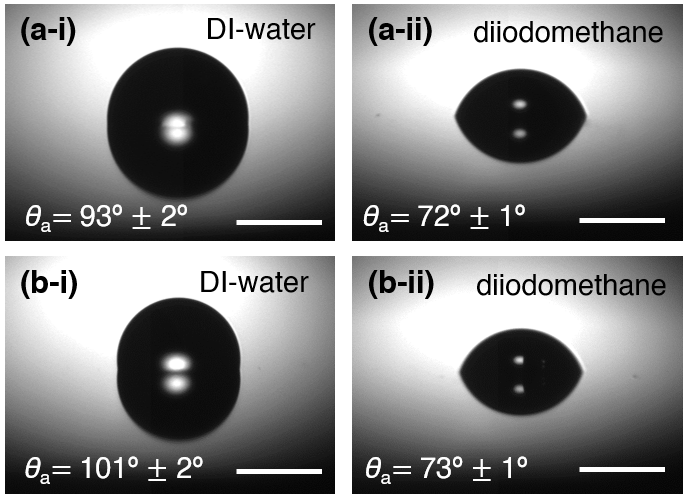


**Supplementary Figure 6.** Surface energy measurements. Side-view optical microscopy images of a deionized water droplet and a diiodomethane droplet residing in the apparent advancing state on the (a) dyn-PDMS (1.5:1), and (b) dyn-PDMS (1.7:1) coated Si-wafer samples, respectively. Each image is labeled with its apparent advancing contact angle, 𝜃_a_. All scale bars represent 1 mm. The uncertainty of the contact angle measurements are determined as the standard deviation of 3 independent measurements at 3 different locations on one single sample.


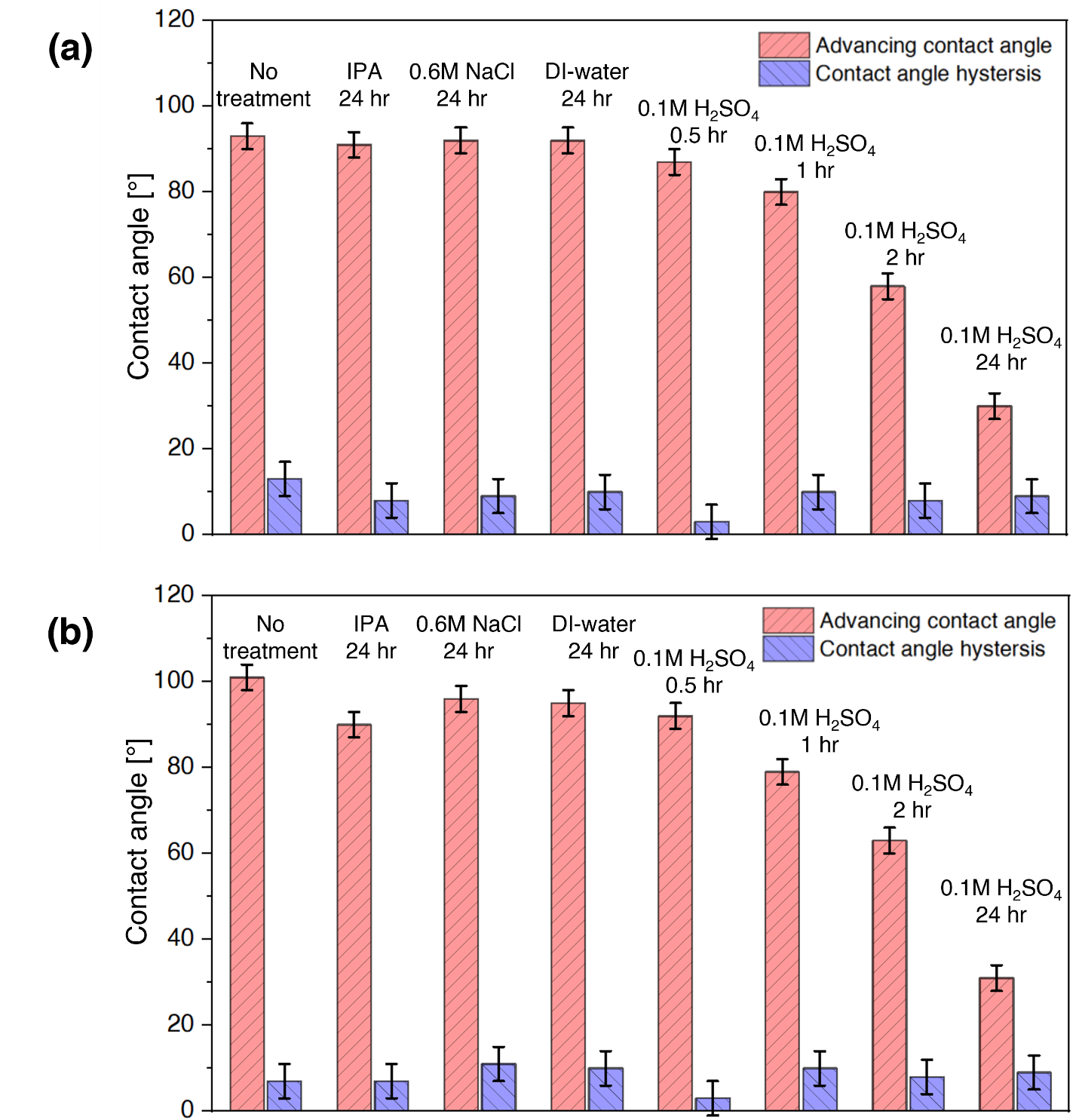


**Supplementary Figure 7.** Chemical robustness of (a) 10 nm 1.5:1 dyn-PDMS and (b) 10 nm 1.7:1 dyn-PDMS samples. Samples were immersed in different liquid environment including IPA, 0.6 mol·L^-1^ NaCl solution, 0.1 mol·L^-1^ H_2_SO_4_ solution, and distilled water. The uncertainty of the contact angle measurements are determined as the standard deviation of 3 independent measurements at 3 different locations on one sample.


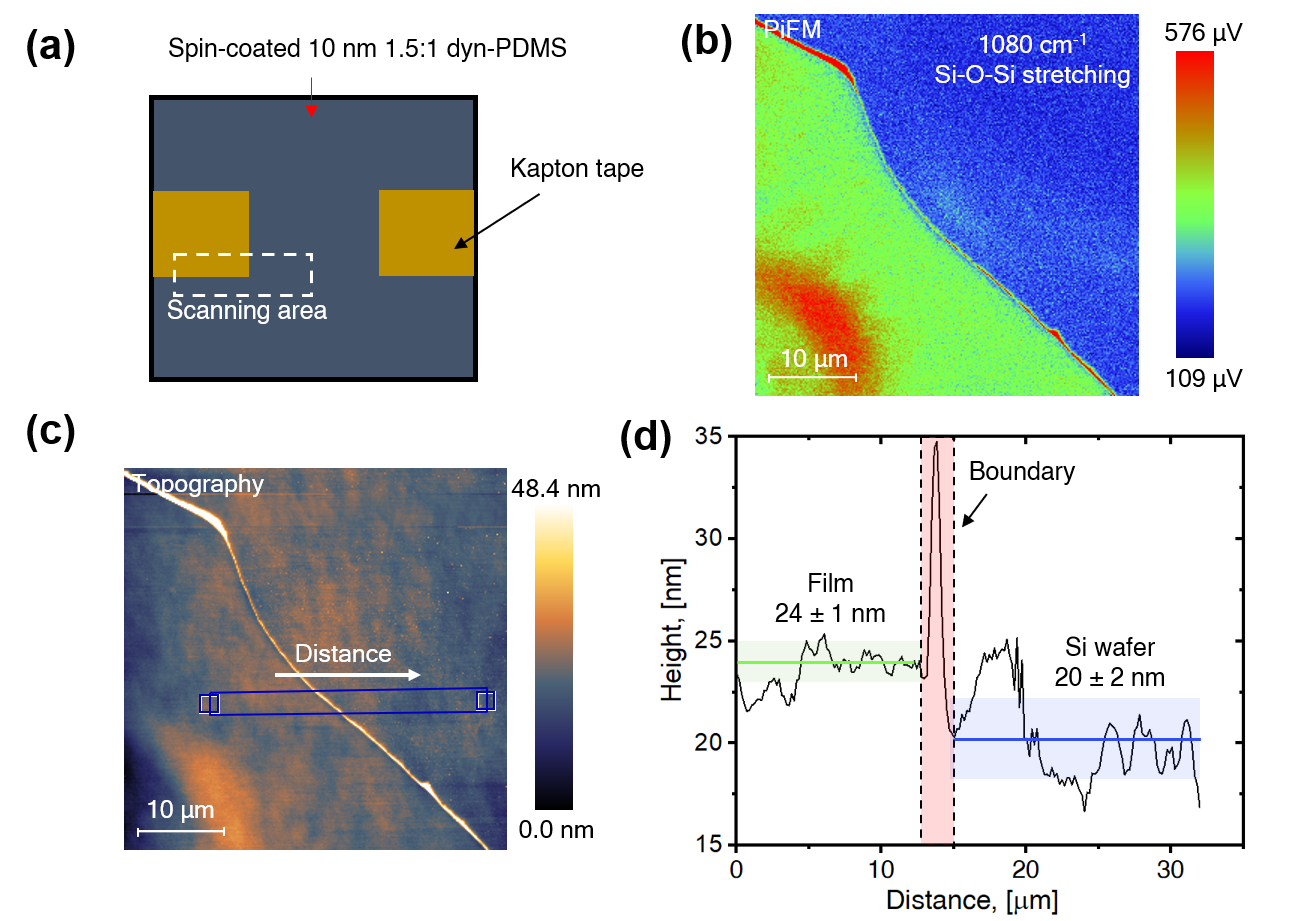


**Supplementary Figure 8.** Coating thickness measurement using AFM-IR step test. (a) Schematic diagram of the sample fabrication process. (b) Absorbance intensity of the 1080 cm^-1^ peak at the boundary of 10 nm dyn-PDMS (1.5:1)film/silicon wafer scanned by AFM-IR. Inset color bar: IR intensity from 109 μV (blue) to 576 μV (red). (c) Morphology of the boundary scanned by AFM-IR. Inset color bar: surface height from low (black) to high (white). (d) The profile of the cut shown in (c) showing the thickness of the film is 4 ± 3 nm. The uncertainty of the height measurements are determined as the standard deviation of the height profile across the whole colored region (Green for the film and blue for the Si wafer, respectively).


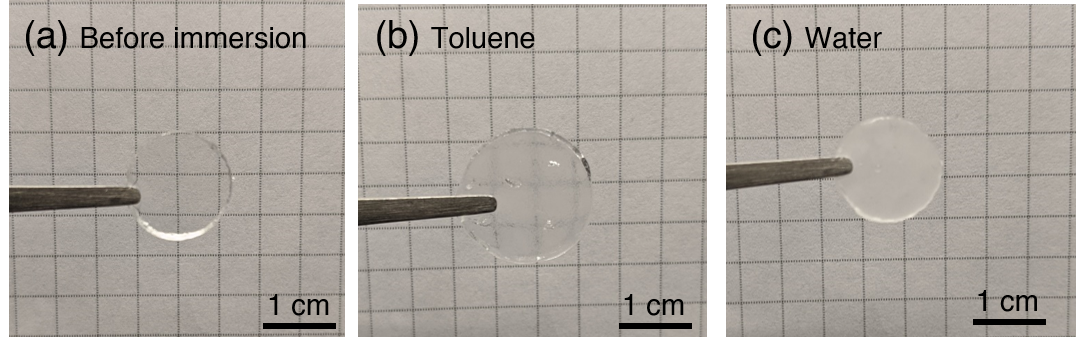
 **Supplementary Figure 9.** Optical images demonstrating swelling of bulk 1.5:1 dyn-PDMS materials (a) before immersion, and after 10 minutes of immersion in (b) toluene and (c) water.


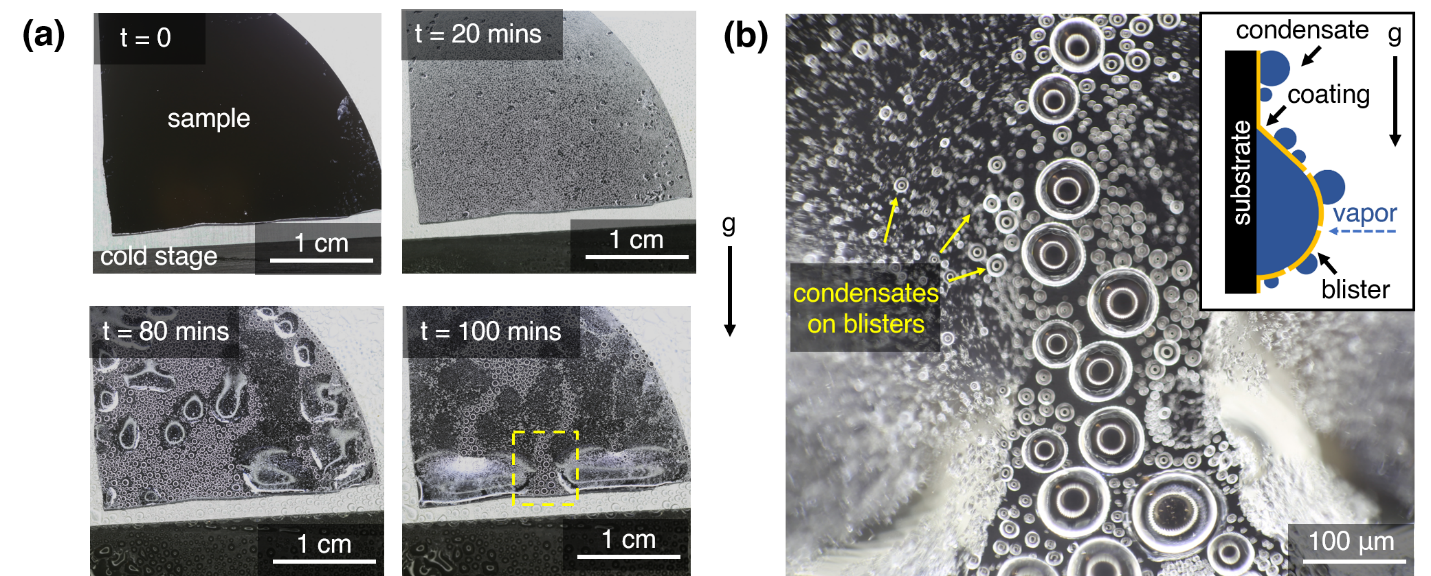


**Supplementary Figure 10.** Optical images demonstrating condensation induced delamination of a 70 nm-thick CF_x_ coating deposited on a polished silicon wafer. (a) Time-lapse optical images of steam condensation on the sample. Water blisters grew beneath the CF_x_ film and resulted in coating delamination. (b) A 10X magnified view of the blistering region as labeled in (a) by the dotted yellow boundary. Spherical condensate droplet residing on top of the blisters were observed. This observation excludes the possibility that the irregular shaped objects are condensate. Insert of (b): Schematic diagram of condensation induced coating delamination (side-view).

**Supplementary Tables**

**Supplementary Table 1.** Surface roughness characterization of all samples reported in Supplementary Figure 4 and 5. The roughness here is defined as the maximum peak-to-peak RMS roughness of the AFM scan. Roughness ratio is defined as the total AFM scan surface area normalized by the AFM scan projected area multiplied by 100%.

| **Coating Materials** | **Substrate** | **Deposition Method** | **AFM Profile** | **Roughness [nm]** | **Roughness Ratio,** $\boldsymbol{r}$ **[%]** |
| --- | --- | --- | --- | --- | --- |
| No coating | Si wafer | N/A | Supp Fig. 4(a) | 0.2 | 100.04% |
|  | Glass |  | Supp Fig. 4(b) | 0.4 | 100.04% |
|  | Aluminum |  | Supp Fig. 4(c) | 17.5 | 102.30% |
|  | Copper |  | Supp Fig. 4(d) | 18.6 | 110.99% |
| Linear PDMS | Si wafer | Spin | Supp Fig. 5(a) | 0.9 | 100.02% |
| dyn-PDMS (1.5:1) | Si wafer | Spin | Supp Fig. 5(b) | 1.1 | 100.03% |
| dyn-PDMS (1.7:1) | Si wafer | Spin | Supp Fig. 5(c) | 0.7 | 100.01% |
| dyn-PDMS (1.5:1) | Si wafer | Dip | Supp Fig. 5(d) | 0.4 | 100.02% |
| dyn-PDMS (1.5:1) | Glass | Spin | Supp Fig. 5(e) | 1.3 | 100.22% |
| dyn-PDMS (1.5:1) | Glass | Dip | Supp Fig. 5(f) | 1.2 | 100.23% |
| dyn-PDMS (1.5:1) | Aluminum | Spin | Supp Fig. 5(g) | 9.3 | 102.55% |
| dyn-PDMS (1.5:1) | Aluminum | Dip | Supp Fig. 5(h) | 3.3 | 100.09% |
| dyn-PDMS (1.5:1) | Copper | Spin | Supp Fig. 5(i) | 28.3 | 105.36% |
| dyn-PDMS (1.5:1) | Copper | Dip | Supp Fig. 5(j) | 15.4 | 100.77% |

**Supplementary Discussions**

**Supplementary Discussion 1. CF_x_ film degradation**

The irregular objects observed in Figure 3a of the manuscript are ‘water blisters’. These blisters also look like condensate films forming on a hydrophilic surface. To show that the irregular objects are indeed water blisters instead of condensate films, we coupled our DSLR camera with a 10X objective (CFI Plan 10X, Nikon). We show the microscopic images of the water blisters in Supplementary Figure 10. We found small condensates form on top of the blister, which indicates there is a layer of solid hydrophobic film between the condensate on top and water beneath. The spherical shape of the top condensate indicates that the film still maintains hydrophobicity after delamination. If the irregular shaped objects were condensate films, it would be impossible to see other condensate droplets residing on top as clearly shown in Supplementary Figure 10.

**Supplementary Methods**

**Supplementary Method 1. Sample thickness measurement by IR-AFM**

To ensure the existence of the 10-nm 1.5:1 dyn-PDMS on the polished silicon wafer, we performed an AFM step measurement. The substrate we prepared was a 3 cm × 3 cm polished silicon wafer. The wafer was first cleaned by rinsing in acetone, IPA, water and IPA in sequence, then dried with a clean N_2_ gas stream. Then, Kapton tape (Purchased from Uline) was partially stuck on to the edge of the silicon wafer (as illustrated in Supplemental Figure 8a). The sample was then further purified through air plasma cleaning (Harrick Plasma, PDC-32G) for ten minutes at high power (RF, 18 W). The 10 nm dyn-PDMS film was then deposited on the cleaned substrate by the spin-coating and annealing process.

Prior to the AFM measurement, the tapes were peeled off manually, and a 40 μm × 40 μm area at boundary of the film and the polished silicon wafer was scanned (Supplemental Figure 8a). The AFM-IR first scanned the IR intensity at 1080 cm^-1^ (Si-O-Si bond stretching peak) across the area, which confirmed the boundary position between the coating (has Si-O-Si bonds, bottom left in Supplemental Figure 8b) and single crystal silicon wafer (limited Si-O bonds, top right in Supplemental Figure 8b). A topology scan was then performed in the same region (Supplemental Figure 8c), where a height profile of the cut was obtained and is shown in Supplemental Figure 8d, which yielded a coating thickness of 4 ± 3 nm. It should be noted that this thickness measurement method has a relatively high noise/signal ratio (80%) compared to ellipsometeric measurements (1%) due to the difficulty in obtain clean cuts, which can be affected by the flow of the materials and residuals after Kapton tape peeling.

**Supplementary Method 2. Surface energy measurements**

The surface energies of a flat surface ($s$) can be measured by the contact angle approach based on the Fowkes model. The model assumes that the surface energy has two components, one is dispersive surface energy $\gamma_{s,d}$ and another is polar component $\gamma_{s,p}$.^1^ The two components of surface energy can be determined by measuring the intrinsic advancing contact angle of water ($\theta_{H_{2}O}$) and diiodomethane ($\theta_{CH_{2}I_{2}}$). All apparent contact angles were measured on at least 3 spatially distinct spots on the surface using a piezoelectric micro-goniometer (MCA-3, Kyowa),^2^ with measurement result summarized in Supplementary Figure 1, along with the uncertainty as defined by the standard deviation of the measurement results. The maximum base radius of the sessile droplets was controlled such that it is smaller than the capillary length (approximately 3 mm) of both water and diiodomethane to ensure the contact angle is not affected by gravity.^2^

The dispersive surface energy, $\gamma_{s,d}$, is measured using diiodomethane (Sigma Aldrich, ReagentPlus, 99%. $\gamma_{CH_{2}I_{2},d}=$50.8 mJ·m^-2^, $\gamma_{CH_{2}I_{2},p}=$ 0 mJ·m^-2^ at 20 ℃) as the probing liquid:

|  | $\gamma_{s,d}=\frac{\gamma_{CH_{2}I_{2}}^{2}\left( 1+cos\theta_{CH_{2}I_{2}} \right)^{2}}{4\gamma_{CH_{2}I_{2},d}}.$ | **(1)** |
| --- | --- | --- |

Then, di-ionized (DI) water ($\gamma_{H_{2}O,d}=$21.8 mJ·m^-2^, $\gamma_{H_{2}O,p}=$ 51.0 mJ·m^-2^ at 20 ℃) was used to obtain $\gamma_{s,p}$:

|  | $\gamma_{s,p}=\frac{\left[ \gamma_{H_{2}O}\left( 1+cos\theta_{H_{2}O} \right)-2\sqrt{\gamma_{s,d}\gamma_{H_{2}O,d}} \right]^{2}}{4\gamma_{H_{2}O,p}} .$ | **(2)** |
| --- | --- | --- |

Contact angle measurements were performed on at least three different spots on each surface, and the uncertainties of contact angle $\Delta\theta_{CH_{2}I_{2}}$ and $\Delta\theta_{H_{2}O}$ are defined as the standard deviation of the different measurements.

The uncertainty of $\gamma_{s,d}$ is calculated by:

|  | $\Delta\gamma_{s,d}=\left\vert\left. \frac{\partial\gamma_{s,d}}{\partial\theta_{CH_{2}I_{2}}}\Delta\theta_{CH_{2}I_{2}} \right\vert\right.,$ | **(3)** |
| --- | --- | --- |

And the uncertainty of $\gamma_{s,p}$ is calculated by:

|  | $\Delta\gamma_{s,p}=\sqrt{\left( \frac{\partial\gamma_{s,p}}{\partial\theta_{H_{2}O}}\Delta\theta_{H_{2}O} \right)^{2}+\left( \frac{\partial\gamma_{s,p}}{\partial\gamma_{s,d}}\Delta\gamma_{s,d} \right)^{2}}.$ | **(4)** |
| --- | --- | --- |

The optical microscopy image of droplet contact angles are included in Supplementary Figure 6. Using the method mentioned above, the surface energy of the 1.5:1 dyn-PDMS is determined to be$\gamma_{s,d}=$ 21.8 ± 0.6mJ·m^-2^, $\gamma_{s,p}=$ 3.1 ± 0.9 mJ·m^-2^. The surface energy of the 1.7:1 dyn-PDMS is determined to be$\gamma_{s,d}=$ 21.3 ± 0.6mJ·m^-2^, $\gamma_{s,p}=$ 1.2 ± 0.7 mJ·m^-2^.

**Supplementary Method 3. Evaluating the chemical robustness of dyn-PDMS thin films**

The chemical robustness of the 10 nm thick dyn-PDMS films (both 1.5:1 and 1.7:1) were empirically tested by measuring the change in apparent advancing contact angle and contact angle hysteresis of deionized-water droplets deposited on samples that have been immerses into different liquid environments (100 mL) for 24 hours. The size of the samples was 1 cm × 1 cm. The liquid used for testing include: organic solvents such as isopropyl alcohol (IPA, CAS # 67-63-0, Fisher Chemical), salt water (0.6 mol·L^-1^ NaCl solution, CAS # 7647-14-5, Millipore Corporation), acid solution (0.1 mol·L^-1^ H_2_SO_4_ solution, pH value $\approx$ 0.7, CAS #7664-93-9, Macron Fine Chemicals), and distilled water (CAS #7732-18-5, Fisher Chemical) as control. During testing, the beakers containing the sample and test liquid were covered by aluminum foil (Reynolds Wrap). The samples immersed in IPA, salt water, and DI-water were taken out after 24 hours of immersion for contact angle measurement. Samples immersed in the 0.1 M H_2_SO_4_ solution were taken out after a fixed intervals of immersion time (0.5 hour, 1 hour, 2 hours and 24 hours) for contact angle measurement. Prior to conducting the measurements, the samples were rinsed with deionized-water then blown dry with a clean N_2_ gas flow. The apparent advancing contact angle and contact angle hysteresis of the samples after immersion are shown in Supplemental Figure 7. The uncertainties of the measurements were determined by considering the standard deviation between three measurements at different locations of one sample.

**Supplementary References**

Condensation of water vapor is a crucial process in many

industries, including power generation and desalination.

Roughly 85% of the global installed base of electricity genera-

tion plants and 50% of desalination plants worldwide rely on

steam condensers.

[

1,2

]

Given the massive scale of these pro-

cesses, any improvements in cycle efﬁ ciencies would have a

profound effect on global energy consumption. Dropwise con-

densation has been an active area of research for nearly a cen-

tury, as the resulting heat transfer coefﬁ cients can be an order

of magnitude higher than those seen in ﬁ lmwise condensa-

tion.

[

3,4

]

However, the practical implementation of this concept

in power generation, desalination, and other applications has

been a signiﬁ cant materials challenge,

[

4

]

limited by durability of

existing hydrophobic functionalization for metal heat transfer

surfaces. While metals provide both high thermal conduc-

tivity for maximizing heat transfer and high tensile strength to

minimize the need for structural supports, metals are typically

wetted by water and most other thermal ﬂ uids and so exhibit

ﬁ lmwise condensation. Thus, metallic heat transfer surfaces

must be modiﬁ ed with a hydrophobic coating to obtain drop-

wise condensation. Previous dropwise promoters have included

self-assembled monolayers of oleic acids,

[

5,6

]

fatty acids, and

also of thin ﬁ lms of polymers applied via sputtering or dip-

coating.

[

7–9

]

However, most of these hydrophobic modiﬁ ers,

and especially the silane-based modiﬁ ers that are ubiquitous

in recent condensation studies, are not robust in steam envi-

ronments of industrial interest. More recent studies have used

nanotextured surfaces to improve condensation heat transfer,

however these surfaces also rely on silane or thiol modiﬁ ers

to switch the wettability of a nanotextured surface from supe-

rhydrophilic to superhydrophobic.

[

10–19

]

Additionally, because

the thermal conductivities of polymeric materials are typically

orders of magnitude smaller than that of a metal substrate, it is

crucial that a polymer modiﬁ er be as thin as possible to mini-

mize thermal resistance. Hence, there is a need for an ultra-

thin robust hydrophobic modiﬁ er.

Condensation of water vapor is a crucial process in many

industries, including power generation and desalination.

Roughly 85% of the global installed base of electricity genera-

tion plants and 50% of desalination plants worldwide rely on

steam condensers.

[

1,2

]

Given the massive scale of these pro-

cesses, any improvements in cycle efﬁ ciencies would have a

profound effect on global energy consumption. Dropwise con-

densation has been an active area of research for nearly a cen-

tury, as the resulting heat transfer coefﬁ cients can be an order

of magnitude higher than those seen in ﬁ lmwise condensa-

tion.

[

3,4

]

However, the practical implementation of this concept

in power generation, desalination, and other applications has

been a signiﬁ cant materials challenge,

[

4

]

limited by durability of

existing hydrophobic functionalization for metal heat transfer

surfaces. While metals provide both high thermal conduc-

tivity for maximizing heat transfer and high tensile strength to

minimize the need for structural supports, metals are typically

wetted by water and most other thermal ﬂ uids and so exhibit

ﬁ lmwise condensation. Thus, metallic heat transfer surfaces

must be modiﬁ ed with a hydrophobic coating to obtain drop-

wise condensation. Previous dropwise promoters have included

self-assembled monolayers of oleic acids,

[

5,6

]

fatty acids, and

also of thin ﬁ lms of polymers applied via sputtering or dip-

coating.

[

7–9

]

However, most of these hydrophobic modiﬁ ers,

and especially the silane-based modiﬁ ers that are ubiquitous

in recent condensation studies, are not robust in steam envi-

ronments of industrial interest. More recent studies have used

nanotextured surfaces to improve condensation heat transfer,

however these surfaces also rely on silane or thiol modiﬁ ers

to switch the wettability of a nanotextured surface from supe-

rhydrophilic to superhydrophobic.

[

10–19

]

Additionally, because

the thermal conductivities of polymeric materials are typically

orders of magnitude smaller than that of a metal substrate, it is

crucial that a polymer modiﬁ er be as thin as possible to mini-

mize thermal resistance. Hence, there is a need for an ultra-

thin robust hydrophobic modiﬁ er.

1. Fowkes, F.M., *Additivity of Intermolecular Forces at Interfaces. I. Determination of the Contribution to Surface and Interfacial Tensions of Dispersion Forces in Various Liquids.* The Journal of Physical Chemistry, 1963. **67**(12): p. 2538-2541.

2. Cha, H., J. Ma, Y.S. Kim, L. Li, L. Sun, J. Tong, and N. Miljkovic, *In Situ Droplet Microgoniometry Using Optical Microscopy.* ACS Nano, 2019. **13**(11): p. 13343-13353.
