# Supplementary material for: Ultra-thin self-healing vitrimer coatings for durable hydrophobicity
Source: Nat Commun. 2021 Sep 1;12:5210. doi: 10.1038/s41467-021-25508-4 (PMC8410847; doi:10.1038/s41467-021-25508-4)
Supplement: Supplementary file 3 — Description of Additional Supplementary Files [file 41467_2021_25508_MOESM3_ESM.docx]

**Description of Additional Supplementary Files**

**Supplementary Video 1.** Steam condensation on a scratched 75 nm thick CF_x_ film (738 μm × 490 μm view, playing at 4× normal speed)

**Supplementary Video 2.** Steam condensation on scratched 10 nm thick dyn-PDMS (738 μm × 490 μm view, playing at 4× normal speed)
